# Socioeconomic status and problem behaviors in young Chinese children: A moderated mediation model of parenting styles and only children

**DOI:** 10.3389/fpsyg.2023.1029408

**Published:** 2023-02-03

**Authors:** Xunyi Lin, Yifan Zhang, Yutong Liao, Wanlin Xie

**Affiliations:** ^1^College of Education, Fujian Normal University, Fuzhou, Fujian, China; ^2^College of Education, Nanchang Institute of Science and Technology, Nanchang, Jiangxi, China

**Keywords:** SES, parenting styles, young children, child number, problem behaviors, moderated mediation

## Abstract

**Introduction:**

This study tested a moderated mediation model of child number (CN) and parenting styles (PS) in the relationships between family socioeconomic status (SES) and young children’s problem behaviors (PB).

**Methods:**

A sample of 1,101 children (M_age_ = 4.90 years, *SD* = 1.07) and their parents participated in this study. Parents reported on PS, SES, and children’s PB.

**Results and Discussion:**

The results show SES was positively related to authoritative parenting and negatively related to authoritarian parenting; problem behaviors were negatively related to authoritative parenting and positively related to authoritarian parenting; authoritative parenting and authoritarian parenting mediated the relationship between SES and PB; and singleton moderated the relationship between SES and PB. The combination of only children and low levels of SES could lead to high PB levels, while the combination of non-only children and high levels of SES could lead to high PB levels. At the same SES, only children had higher PB levels than non-only children.

## 1. Introduction

China’s population policy has been in the process of constant updating and adjustment to an aging population. After the one-child policy was initiated in 1980, China’s unique “sandwich family structure” gradually emerged (Gong et al., 2016), with adults expected to care for the elderly and the young. As China began implementing its two-child and three-child policies, family structures transformed from ones with only one child to ones including siblings (Padmadas et al., 2017). Sibling relationships have a significant and lasting impact on children’s development (Wolke et al., 2015). On the one hand, children may benefit directly from the learning, company, and affection siblings can provide (Rochebrochard and Joshi, 2013); on the other, siblings could be a liability. For example, there is increasing evidence that sibling bullying, a form of repeated aggression, adversely affects children’s mental health and triggers behavioral problems (Wolke et al., 2015). Parents play a crucial role in avoiding this situation by educating their multiple children and providing good parenting to buffer children’s negative behaviors.

Many factors influence parenting styles and children’s problem behaviors, including social-economic status. According to family system theory, the family is conceptualized as a system in which family members and relationships influence each other directly and indirectly (Ensminger and Fotherill, 2003). The previous study has found that social-economic status affects families’ and individuals’ functioning through various stress and investment processes (Conger and Donnellan, 2007). Besides that, many studies have revealed the influences of parenting and siblings on young children’s development, but little is known of how these factors interactively affect child problem behaviors (Gibbs et al., 1987; Teti and Ablard, 1989; Stormshak et al., 2010; Lipina et al., 2013; Dolean et al., 2019; Kracht et al., 2019). To fill the knowledge gap, we explored how social-economic status influences children’s problem behaviors by examining the role of parenting styles and the number of children in the family. The results of this study most importantly revealed that singleton could moderate the mediation relationships between family socio-economic status, different parenting styles, and children’s problem behaviors.

### 1.1. Socioeconomic status and child development

Socioeconomic status has long played a central role in social and developmental sciences (Ensminger and Fotherill, 2003). The measures of social-economic status are typically comprised of three indicators, family income, parents’ education and parents’ occupation (Rindermann and Baumeister, 2015). Social-economic status is not only an important factor in determining the placement of families within the social hierarchy (Daphne et al., 2019), but also a powerful predictor of many aspects of child development and well-being (Hoff, 2010; Tonizzi et al., 2020). Children from families with higher SES can experience a better quality of literacy and other aspects (Irwin et al., 2007). However, children from socio-economically disadvantaged backgrounds demonstrate lower performance on short-term memory, inhibition and reading-related factors (Lipina et al., 2013; Dolean et al., 2019; Sheridan et al., 2020), since the financially stressed families are less able to provide the tangible or intangible resources necessary to support their children’s successful development (Bradley and Corwyn, 2002).

Family environment is another strong determinant of children’s development (Peter et al., 2018), especially in Chinese contexts (Yiu et al., 2021). Focusing on the family environment is natural because it is the social context in which children typically spend most of their time and have many key relationships (Barber and Olsen, 1997). The family environment is the sum of physical and psychological conditions, which carries the development of individual personality and behavior (Zhao and Zhao, 2022). According to the family systems theory, the family is composed of several subsystems, which are interconnected and mutually constrained to make the whole family function well, and the better the coordination of the family system, the better the psychological shape and academic performance of the members (Miller et al., 1985). Research on typical child development has extensively and consistently demonstrated the family environment’s effects on children’s language and behavior pullulation (Mcgillicuddy-Delisi, 1983; Soliday et al., 2001; Hoff, 2003). For example, children reared in family environments characterized by marital discord especially parental conflict, are more aggressive than children from comparison families (Halpern, 2004). Bronfenbrenner (1983) ecological theory suggests that social class differences in the microenvironment influence children’s development but does not explain why the differences exist (Bradley et al., 1989). Given that social-economic status is often confounded with such environmental variables as paternal absence, degree of crowding within the home, birth order, and so forth (Zajonc, 1976), it would be meaningful to explore how social-economic status is reflected in the family environment first and then affects children’s development (Bradley and Caldwell, 2015). Casey et al. (1984) suggested that low social-economic status families are more likely to have less healthy family environments, leading to situations that are not conducive to child behavioral development.

### 1.2. Socioeconomic status, parenting styles, and children’s problems behaviors

Problem behavior in children can be manifested in either externalizing or internalizing behavior (Aunola and Nurmi, 2005). The link between social-economic status and children’s problem behaviors has received little attention from researchers before. Recently, more scholars have begun to pay attention to this problem. Some researchers found social-economic status affects families’ and individuals’ functioning through various stress and investment processes (Conger and Donnellan, 2007). For example, a low social-economic status family may have more family conflict, reduced marital warmth, and diminished parenting qualities, thus affecting parenting styles and jeopardizing child development (Wang et al., 2021). Besides, some researchers hypothesized that low and unstable social-economic status over the first 10 years would relate to more child behavior problems, noting that children whose families had low social-economic status were at greater risk of having internalizing behaviors during their first 10 years than those in middle social-economic status families (Sim et al., 2012). The results of this study did not find the relationship between social-economic status and children’s problem behaviors. This problem needs to be addressed in our future research.

Parental socialization refers to the process by which the adult can transmit to the young person the habits and values of the culture of origin so that the child adopts adequate functioning within the culture to which the child belongs (Villarejo et al., 2020; Climent-Galarza et al., 2022). Of the many parental socialization variables, parenting styles are among the most frequently investigated (Steinberg, 2001), with several studies examining their role in children’s internalizing and externalizing problem behaviors (Hart et al., 2003; Alizadeh et al., 2011; Berkien et al., 2012; Shafipour et al., 2015). Parenting styles refer to the typical ways parents think, feel and behave in terms of child-rearing (Levin, 2011), which captures two important elements of parenting: strictness and warmth. Strictness refers to the degree to which parents try to control their children’s behavior by setting rational standards for behavior. This parenting dimension has been labeled in different ways as control (Jastrow, 1929; Schaefer and Earl, 1959), firm control (Steinberg, 2005), demandingness or authority (Maccoby and Martin, 1983), more recently called strictness or imposition (Climent-Galarza et al., 2022). Warmth refers to the amount and way love is expressed to the children and acceptance of the children’s points of view. This parenting dimension has been called acceptance or involvement (Lamborn et al., 1991; Chao, 2001) and affection or responsiveness (Maccoby and Martin, 1983). Both dimensions have been associated with children’s problem behavior (Aunola and Nurmi, 2005). A meta-analytic review involving 1,435 studies of parenting and child problem behaviors showed that punitive parenting practices and psychological control were likely to trigger children’s problem behaviors (Pinquart, 2017). Conversely, a warm relationship between parents and children has been shown to reduce children’s emotional and behavioral problems (Lansford, et al., 2014).

The three categories of parenting styles based on these dimensions and accepted by most researchers are proposed by Baumrind (1971), including authoritarian, authoritative, and permissive parenting. Authoritarian parents are used to controlling the behavior and attitudes of children, they value obedience as a virtue and favors punitive and expect their orders to be obeyed without explanation (Santrock, 2011). On the contrary, the authoritative parents attempt to direct the child’s activities in a rational, issue-oriented manner. These parents are more responsive to their children and allow for an open dialog and space for questioning authority (Baumrind, 2012). Permissive parenting, wherein parents are least demanding of their children. They do not expect much from their children in terms of maturity and self-control and fail to discipline them (Baumrind, 1971). Maccoby and Martin (1983) expanded Baumrind’s permissive parenting style into two different parenting types: permissive style (also referred to as indulgent parenting style) and neglectful parenting (also referred to as uninvolved parenting style). Children’s freedom and autonomy are highly valued in permissive parenting family, and parents tend to rely mostly on reasoning and explanation. An uninvolved or neglectful parenting style is when parents are often emotionally absent and sometimes even physically absent.

The two-dimensional model and four types of parenting styles triggered many discussions and researches. Many researchers have suggested that parenting style variables contribute less to children’s adjustment separately than in certain combinations (Baumrind, 1991). And the link between parenting styles and problem behavior varies widely across cultures. For instance, characterized by a high level of parental affection and behavioral control, the authoritative parenting in Anglo-Saxon contexts with European-American samples (mostly white middle-class families) is positively associated with adjustment in children of various ages (Baumrind, 1966; Lamborn et al., 1991; Laurence et al., 1994). However, the authoritarian parenting was associated with the best child adjustment in other studies conducted in Arabs societies (Dwairy, 2006) and in ethnic minority groups in the United States such as Chinese Americans (Chao, 2001) or African American (Deater-Deckard et al., 1996). While the most recent research, conducted in European and Latin American countries, indicates that children from indulgent families show greater adjustment on different adjustment criteria including self-concept, satisfaction with life, empathy and psychosocial maturity (Garcia et al., 2020; Perez-Gramaje et al., 2020).

### 1.3. The role of child number

The existing literature mainly focuses on the relationships between social-economic status and children’s development, whereas limited research pays attention to the important influence of family structures and sibling relationships between social-economic status and children’s development. The Family Investment Model (FIM) claims that parents with higher social-economic status have more social capital to promote their children’s greater development (Roubinov and Boyce, 2017). China has begun to implement the two-child and three-child policies. However, affected by many aspects such as the high cost of raising a child and the changing of the Chinses traditional thinking of “raising children to provide against old age,” the fertility desires of middle and above-income families from China seem to be low (Eklund, 2016; Li et al., 2021). Therefore, lower social-economic status families may have higher fertility desires thus forming multi-child family structures. For those low social-economic status families, sibling resource sharing can reduce the average costs of each child, and then parents may invest more money in children’s education to promote their children’s development with better educational environments. Besides that, according to attachment theory, children’s internal working model of relationships shapes their behaviors in subsequent relationships, such as the sibling relationship (Teti and Ablard, 1989). The sibling subsystem provides a unique and powerful influence that can promote, detract from, or be independent of parents’ efforts to socialize their children (Brody, 1998). The first study of older siblings’ contributions to their younger brothers’ and sisters’ development were conducted in Britain around the turn of the 20th century (Brody, 2004). Since then, parents and researchers in developmental psychology have gradually recognized the sibling relationship’s significant contribution to children’s development. Many studies have demonstrated that sibling relationships could contribute to children’s cognitive, linguistic, social, emotional, and healthy development (Gibbs et al., 1987; Gass et al., 2007; Stormshak et al., 2010; Kracht et al., 2019). For example, Stormshak et al. (2010) found that sibling relationships provide one of the most stable and powerful developmental contexts for the transmission of prosocial behavior, which can build children’s competence in self-regulation and emotional understanding (Stormshak et al., 2010). The above literature shows that children with siblings are less likely to develop problem behaviors.

### 1.4. The present study

**Figure 1:**
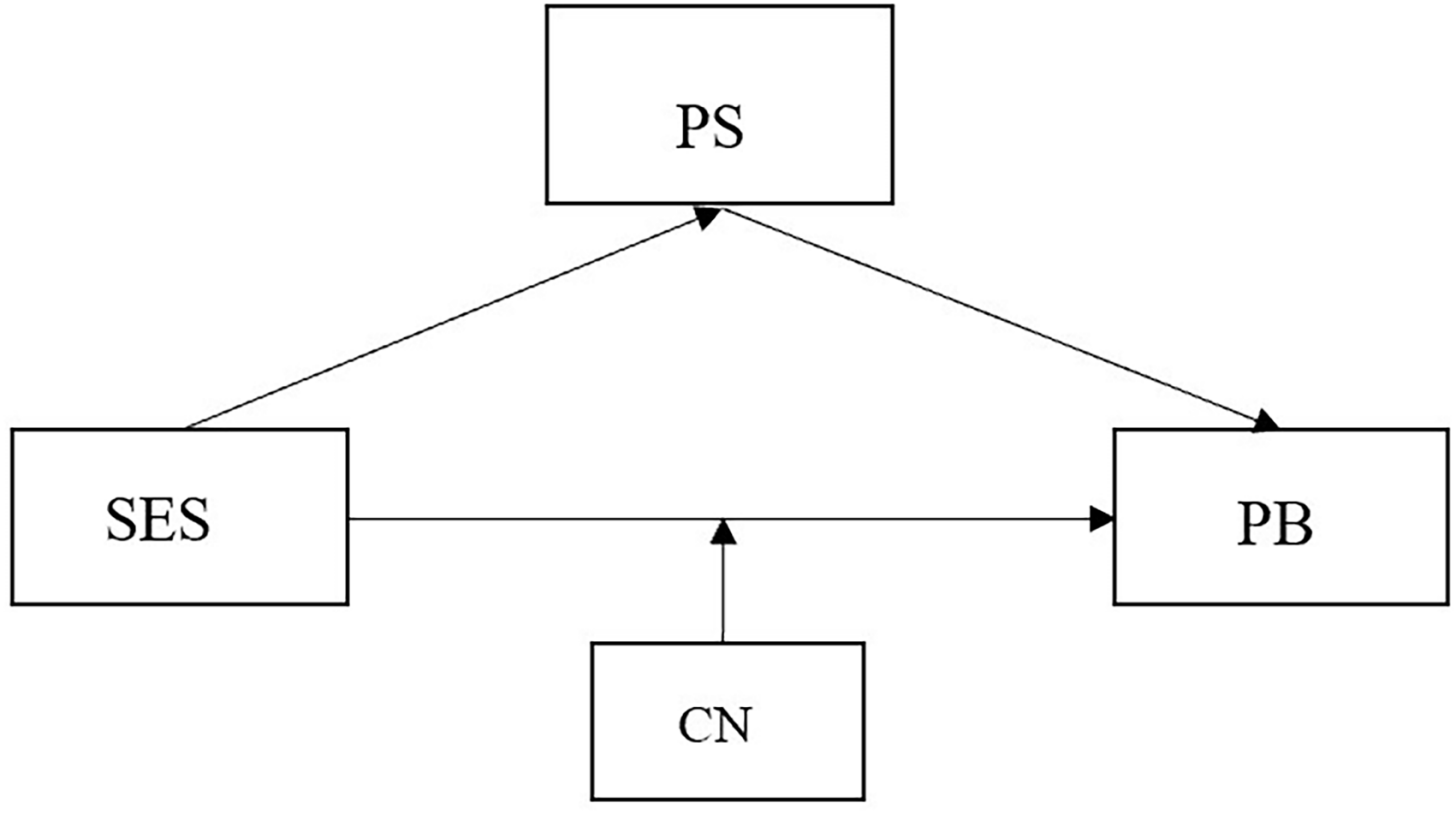
Theoretical model. SES, socioeconomic status; PS, parenting style; PB, problem behaviors; CN, child number.

This paper explores the relationship between social-economic status, problem behaviors, patenting styles and child number. As the literature review revealed that social-economic status could impact problem behaviors through the family environment, which includes factors like parenting styles and family member, we took social-economic status as the independent variable, problem behaviors as the dependent variable, and used parenting styles and child number as intermediate variables. In this setting, we design a mediating model (Figure 1) that can clearly show how social-economic status affects problem behaviors through parenting styles and child number.

Accordingly, the following specific hypotheses are tested in this study:

*Hypothesis 1*: Social-economic status would be negatively related to problem behaviors (H1a). Social-economic status would be positively related to authoritative parenting (H1b) and negatively related to authoritarian parenting (H1c).

*Hypothesis 2*: Problem behaviors would be negatively related to authoritative parenting (H2a) and positively related to authoritarian parenting (H2b).

*Hypothesis 3*: Authoritative parenting and authoritarian parenting would mediate the relationship between social-economic status and problem behaviors.

*Hypothesis 4:* Singleton would moderate the relationship between social-economic status and problem behaviors (H4a). At the same levels of social-economic status, only children would have higher problem behaviors levels than non-only children do (H4b).

## 2. Materials and Methods

### 2.1. Participants and procedure

Participants (1,101 children—529 girls and 672 boys—and their parents) were recruited from three kindergartens in Fuzhou, a large city in southeastern China. Most parents (61.6%) held a bachelor’s degree or higher, while 17.3% of fathers and 17.5% of mothers had a high school education or lower. Monthly family income was divided into three levels: under RMB7,000 (6.9%); RMB7,000 to RMB 24,999 (66.9%); and over RMB25,000 (26.2%). The results indicated most participants were from middle-and high-SES families. Table 1 presents the participants’ demographic information.

**Table 1:** Participant characteristics (*N* = 1,101).

Demographic characteristics	Frequency (%)
Child age in month (M ± *SD*)	58.8 ± 12.9
**Child gender**	
Female	529 (48.0)
Male	672 (57.2)
Child number	
Only children	419 (38.1)
None-only children	682 (61.9)
**Paternal education**	
High school and below	190 (17.3)
Associated degree	233 (21.2)
Bachelor degree	511 (46.4)
Master degree and above	167 (15.2)
**Maternal education**	
High school and below	193 (17.5)
Associated degree	230 (20.9)
Bachelor degree	556 (50.5)
Master degree and above	122 (11.1)
**Household income**	
Low (<6,999 RMB per month)	75 (6.8)
Medium (≥7,000 and <24,999 RMB per month)	737 (66.9)
High (≥25,000 RMB per month)	289 (26.2)

The numbers in the brackets represent the percentage of the sample. High, medium and low household incomes are based on top quartile, middle half and bottom quartile of the urban population in Fujian (Chunhong and Yang, 2018).

The subjects were recruited from three kindergartens with different quality levels in Fuzhou, a coastal city in Southeast China. After being told the purpose of this study, parents first completed the questionnaires [a demographic form, a Children’s Behavior Evaluation (CBE) based on their children’s behaviors, and then filled out a Parenting Style and Dimension Questionnaire (PSDQ)] *via* the Internet. Informed consent was obtained at the start of the survey; all involved parents were advised that their participation was purely voluntary and they could withdraw at any time. The online survey took approximately 15 min to complete. Two researchers downloaded the completed questionnaires and analyzed the data.

### 2.2. Measures

#### 2.2.1. Demographics

The demographic questionnaire collected background information from the participants. The first part dealt with children’s information, including age and gender; the second collected parents’ education and family income data.

#### 2.2.2. Parenting styles.

Robinson et al. (1995) developed the Parenting Style and Dimension Questionnaire (PSDQ), which has been tested to be applicable in both Western (Robinson et al., 1995) and Chinese contexts (Fu et al., 2013). It is a 32-item self-report measure that assesses parenting styles in accordance with Baumrind’s (1991) typologies of parenting styles. Its authoritative parenting style scale (Cronbach’s alpha = 0.86) includes 15 items and three sub-projects. Connection (warmth and support) means parents being supportive, understanding and responsive to children’s feelings or needs. The topics included are “Aware of problems or concerns about child in school” or “Gives praise when child is good” and so on. Adjustment (rules) is of regulation dimension, which means whether the parents will explain or emphasizes reasons to children. Topics in adjustment include such as “Gives child reasons why rules should be obeyed” or “Emphasizes the reasons for rules.” Autonomy (democratic participation) dimensions means whether parents willing to consider their children’s ideas and give them the opportunity to make decisions about certain things including items like “Allows child to give input into family rules” or “Takes into account child’s preferences in making family plans.” Its authoritarian parenting style scale (Cronbach’s alpha = 0.82) comprises 12 items and three sub-projects. The corporal punishment means using physical punishment as a way of disciplining children, containing items such as “Uses physical punishment as a way of disciplining our child” or “Spanks when our child is disobedient.” Hostile speech indicates yelling or shouting to children, which comprises descriptions “Explodes in anger towards child” or “Yells or shouts when child misbehaves.” And arbitrary punishment dimensions points to punishing children without reasons or explanations, including items such as “Punishes by taking privileges away from child with little if any explanations” or “Uses threats as punishment with little or no justification.” These two subscales used a five-point scale ranging from 1 (never) to 5 (always). In this study, Cronbach’s alphas for authoritative and authoritarian parenting were 0.91 and 0.81, respectively.

#### 2.2.3. The children’s behavior evaluation

Part of the Social Competence and Behavior Evaluation, the Children’s Behavior Evaluation (CBE) evaluates children’s problem behaviors (LaFreniere and Dumas, 1996). Its two subscales—anger-aggression (Cronbach’s alpha = 0.66) and anxiety-withdrawal (Cronbach’s alpha = 0.81)—use six-point scales ranging from 1 (never) to 6 (always). The scale has been validated as appropriate in the Chinese context (Qin and Yong, 2002). The anger-aggression scale utilizes anger and aggression levels to evaluate children’s externalizing problem behaviors. There are items such as “Be irritable and lose temper easily” and “Conflict with other children easily.” The anxiety-withdrawal scale covers aspects of depression, anxiety, and isolation to mirror children’s internalizing problem behaviors. This scale includes following topics like “Usually stay alone” or “Easily frustrated.” Young children with a high score on these two scales are more likely to have problem behaviors. In this study, the Cronbach’s alphas for the anger-aggression and anxiety-withdrawal subscales were 0.75 and 0.87, respectively.

#### 2.2.4. Socioeconomic status

social-economic status (SES) is a comprehensive indicator that can be synthesized in many ways (Ren, 2010). Based on previous research, social-economic status in this study includes two main indicators: parental education and family income (e.g., Prus, 2007; Guo et al., 2018). Factor analysis is a usual means of constructing a social-economic status index (Naeeim and Rahman, 2018). Using SPSS, we calculated the standard values of parental education and family income, conducted factor analyses on both, and used the synthesis score to determine each family’s social-economic status.

### 2.3. Data analysis

Descriptive analysis was first conducted within all variables of the study. Structural equation modeling (SEM) was then carried out to test the hypothesized moderated mediation model that specifies the relationship among SES, parenting styles, child number, and child problem behaviors using Mpus 7.0. SEM was evaluated using the Maximum Likelihood method of parameter estimation. The Indirect effect was evaluated by bootstrapping procedures (Preacher and Hayes, 2008), and the following indices were used to test the hypothetical model’s data fit: the chi-square test (χ2), the Root Mean Square Error of Approximation (RMSEA < 0.08), the Standardized Root Mean Square Residual (SRMR < 0.10), the Tucker-Lewis Index (TLI > 0.95), and the Bentler’s comparative Fit Index (CFI > 0.95).

## 3. Results

### 3.1. Descriptive statistics

The descriptive statistics of all variables, including SES (socio-economic status), PS (parenting styles), CN (child number), and PB (problem behaviors), are displayed in Table 2. Results showed that the mean problem behaviors score was positively related to authoritarian parenting (r = 0.324, *p* < 0.001) and negatively related to authoritative parenting (r = −0.232, *p* < 0.001). Results also indicated that problem behaviors were negatively related to child number (r = −0.100, *p* < 0.01) and SES (r = −0.232, *p* < 0.001). Additionally, socio-economic status was significantly related to child number (r = −0.099, *p* < 0.01), authoritative parenting (r = 0.282, *p* < 0.001), and authoritarian parenting (r = −0.107, *p* < 0.001). However, there was no significant relationship between socio-economic status and problem behaviors.

**Table 2:** Means, standard deviations, and intercorrelations of variables in this study.

	M	SD	1	2	3	4	5
1. Child number	—	—	1				
2. SES	—	—	−0.099^**^	1			
3. Authoritative parenting	4.126	0.548	−0.087^**^	0.282^***^	1		
4. Authoritarian parenting	1.638	0.354	0.006	−0.107^***^	−0.0410^***^	1	
5. Problem behaviors	2.195	0.578	−0.100^**^	0.017	−0.232^***^	0.324^***^	1

^*^p < 0.05; ^**^p < 0.01; ^***^p < 0.001. The standardization index of SES was calculated by factor analysis in this study.

### 3.2. Moderated mediation model

**Figure 2:**
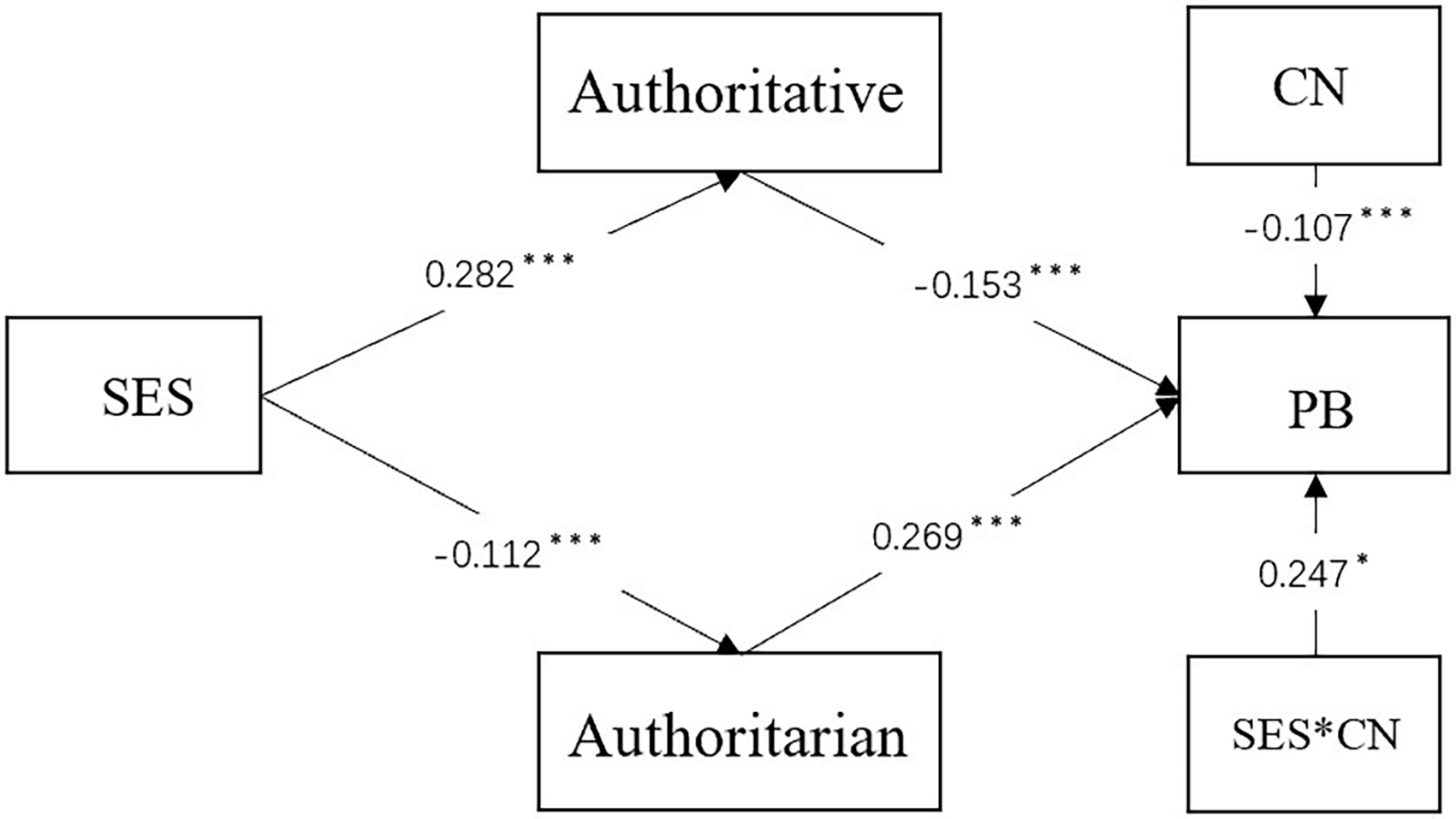
The moderated mediation model. *N* = 1,101. ^∗^*p* < 0.05, ^∗∗^*p* < 0.01 and ^∗∗∗^*p* < 0.001. SES, socioeconomic status; PB, problem behaviors; CN, child number.

The model examined the associations between socio-economic status, child number, authoritative parenting, authoritarian parenting, and problem behaviors (see Figure 2). Mplus analysis proved that the moderated mediation model had a good model fit. χ2 = 5.910 (*N* = 1,101), CFI = 0.996, TLI = 0.987, RMSEA = 0.021, SRMR = 0.012. Bootstrapping of 2000 samples was used to identify the indirect effect with a 95% CI not containing zero. Results showed that authoritative parenting (β = −0.043, SE = 0.012, *p* < 0.001, 95%CI = [−0.014, −0.006]) and authoritarian parenting (β = −0.029, SE = 0.010, *p* < 0.01, 95%CI = [−0.010, −0.003]) mediated the relationship between socio-economic status and problem behaviors, supporting H3. socio-economic status’s direct effect on problem behaviors was not significant (see Table 3).

**Table 3:** Structural coefficients of the final model.

		Coefficient	SE	*P*	95%CI	
PB	SES	−0.161	0.104	0.121	−0.343	0.004
	Authoritative	−0.153	0.046	0.000	−0.214	−0.094
	Authoritarian	0.269	0.037	0.000	0.206	0.0326
	CN	−0.112	0.029	0.000	−0.158	−0.065
	SES*CN	0.247	0.104	0.018	0.077	0.425
Authoritative	SES	0.282	0.031	0.000	0.227	0.329
Authoritarian	SES	−0.107	0.033	0.001	−0.162	−0.056

*N* = 1,101, CN, child number; PB, problem behaviors; SES, socioeconomic status.

**Figure 3:**
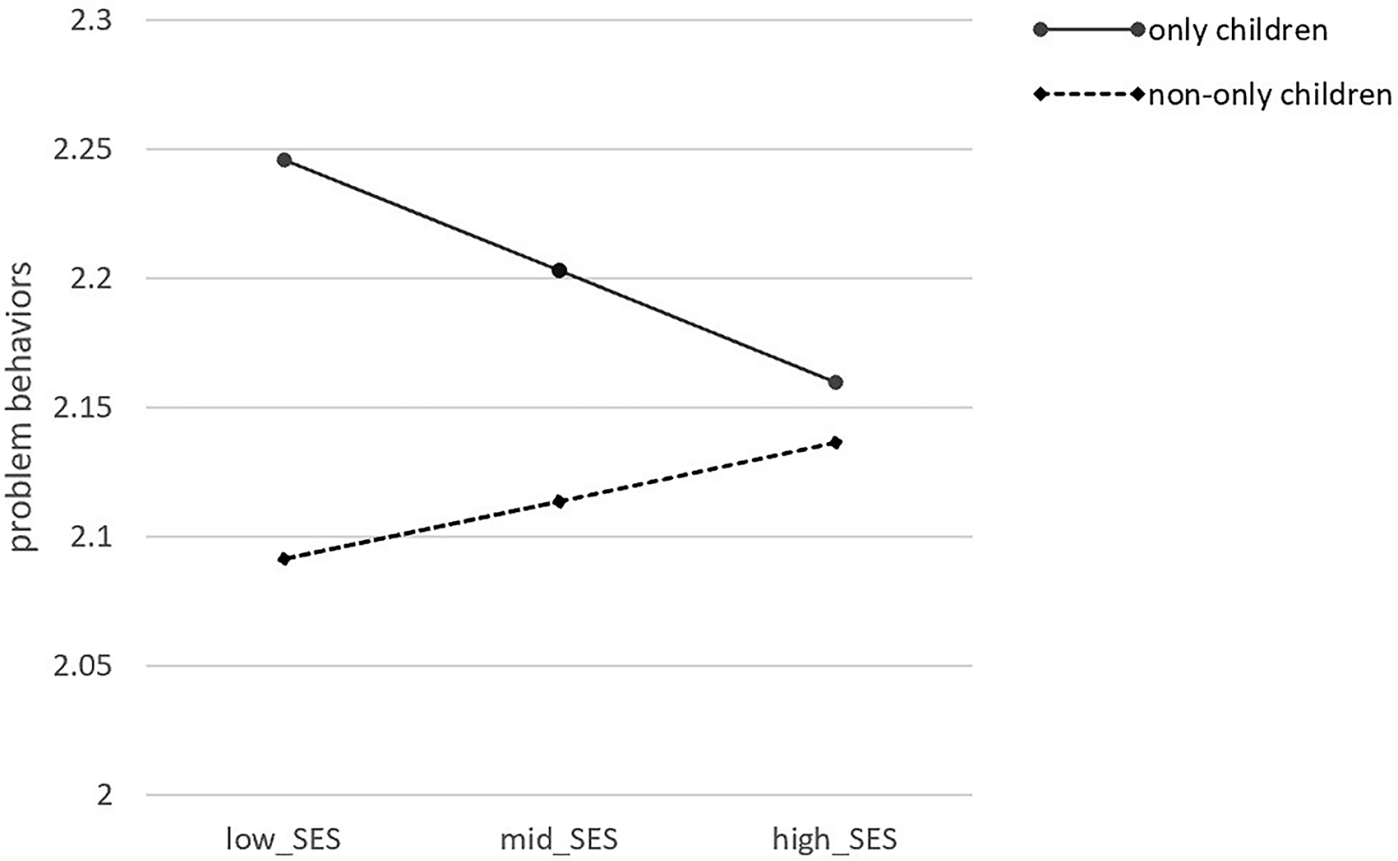
Conditional Effects of the Focal Predictor (SES) at Values of the Moderator as only child number. *N =* 1,101. Mean change is displayed separately for only children and non-only children.

Additionally, the interaction of socio-economic status and child number significantly influenced PB (β = 0.247, t = 2.368, *p* < 0.05), supporting H4a (see Table 3). We explained this significant interaction *via* a simple slope (Figure 3). As the figure shows, the combination of only children and low socio-economic status could lead to high problem behaviors levels; in contrast, the combination of non-only children and high socio-economic status could lead to high problem behaviors levels. In addition, at the same socio-economic status level, only children might have higher problem behaviors levels than non-only children, supporting H4b.

## 4. Discussion

The goal of this study is to find out the links between family socioeconomic status, children’s problem behaviors, patenting styles and child number. Based on this, we proposed four hypotheses and elaborated them separately. Through per path analysis, this study revealed that socio-economic status could significantly predict both authoritative parenting and authoritarian parenting. Additionally, we found a negative relationship between problem behaviors and authoritative parenting, and a positive relationship between problem behaviors and authoritarian parenting. Our analysis also revealed that authoritative parenting and authoritarian parenting could mediate the relationship between socio-economic status and problem behaviors, and child number could moderate the relationship between socio-economic status and problem behaviors.

Our first hypothesis is social-economic status would be negatively related to problem behaviors (H1a), positively related to authoritative parenting (H1b) and negatively related to authoritarian parenting (H1c). We found a significantly positive correlation between socio-economic status and authoritative parenting and a negative correlation between socio-economic status and authoritarian parenting, partially supporting H1. Parenting differs across socioeconomic strata (Hoff and Laursen, 2019). Many empirical studies confirm that low socio-economic status families show higher levels of confused and unstable routines than high socio-economic status families (Evans et al., 2005; Fiese et al., 2013). Parents with lower socio-economic status tend to act harsher and be more punitive (Hoffman, 2003). We found no significant direct effect between socio-economic status and problem behaviors in the present study, inconsistent with the study of Granero et al. (2015). In line with Bronfenbrenner’s theory, young children’s development is affected by the interaction of the exosystem (SES) and the microsystem (PS).

The second hypothesis is problem behaviors would be negatively related to authoritative parenting (H2a) and positively related to authoritarian parenting (H2b). More recent studies confirm that complex models of family contextual factors shape children’s development, indicating that the correlation between socio-economic status and problem behaviors might be influenced by other family environmental factors, such as parenting style and family structure (Roubinov and Boyce, 2017; Luo et al., 2019). Supporting H2, we found that problem behaviors were negatively related to authoritative parenting and positively related to authoritarian parenting, corroborating existing studies (Baumrind, 1991; Padilla-Walker et al., 2010). Authoritarian parenting with high demand and low response is more likely to lead to young children’s misbehaviors (Rinaldi and Howe, 2012). These findings disagree with other recent studies conducted in European and Latin American countries in which indulgent parenting is related to the best child adjustment (Garcia et al., 2020; Perez-Gramaje et al., 2020).

The third hypothesis is authoritative parenting and authoritarian parenting would mediate the relationship between social-economic status and problem behaviors. The results revealed authoritative parenting and authoritarian parenting could mediate the relationship between socio-economic status and problem behaviors, consistent with Luo et al.’s (2019) study and supporting H3. More specifically, parents with high socio-economic status tend to be more authoritative, providing young children with an autonomous environment and emotional support (Williams et al., 2009). Such parenting can reduce young children’s possibility of developing problem behaviors. In contrast, low socio-economic status parents exhibit harsher practices and offer little emotional attention (Hoffman, 2003), resulting in tense parenting and young children’s negative emotions and problem behaviors.

The last hypothesis is child number would moderate the relationship between social-economic status and problem behaviors (H4a), only children would have higher problem behaviors levels than non-only children do at the same levels of social-economic status (H4b). The study reveals that child number significantly moderated the effect between socio-economic status and problem behaviors, supporting H4a. The combination of only children and low socio-economic status could lead to high problem behaviors levels, while the combination of non-only children and high socio-economic status could lead to high problem behaviors levels. Previous research has documented the significance of family structure and sibling relationships in shaping young children’s developmental outcomes (MacKinnon, 1989). The Family Investment Model (FIM) holds that parents with higher socio-economic status have more social capital to promote their children’s greater development (Roubinov and Boyce, 2017). Shi et al. (2021) showed that only children in rural China are in more unfavorable conditions than non-only children due to low socio-economic status family contexts and insufficient educational resources. The average cost of raising only children could be higher than for raising non-only children (Chen, 2010). In low socio-economic status families, multi-child family structures contribute to sibling resource sharing, and parents may invest more money in children’s education (Qian, 2009). By comparison, only children from low socio-economic status families are at greater risk of disadvantageous developmental outcomes—partly because of a lack of cross-age sibling interactions and partly because their parents’ investment in education could be limited by the higher costs of raising only children (Shi et al., 2021). Only children might have higher problem behaviors levels than non-only children at the same socio-economic status level, supporting H4b. Many empirical studies indicate that only and non-only children have different developmental situations (Jiao et al., 1986; Falbo and Polit, 1993). Sibling interaction is important in promoting young children’s social competence (Broderick, 1995). Therefore, only children are in an inferior social development position due to a shortage of sibling interactions and probably engage in higher levels of problem behavior (Lamarche et al., 2006; Padilla-Walker et al., 2010).

## 5. Limitations, future directions, and implications

This study still has some limitations. First, all study variables were assessed using questionnaires completed by parents, meaning that participants may periodically respond to queries in a given direction, amplifying obvious results. To avoid this phenomenon, future studies should examine socio-economic status and children’s problem behaviors using observational and direct assessment methods. Additionally, the study was conducted in cities along China’s eastern coast, where most families have an upper-middle socio-economic status; as such, caution must be exercised in promoting our findings. Future studies should broaden the scope of participants to make the findings more relevant.

Despite these limitations, this study has theoretical implications. We examined a model that investigates parenting styles and child number in a family as mediating and moderating mechanisms between family socio-economic status and young children’s problem behaviors. This study revealed a significantly positive correlation between socio-economic status and authoritative parenting, and a negative correlation between socio-economic status and authoritarian parenting. This study also has practical implications. It further examined how socio-economic status affects children’s problem behaviors through parenting styles and the moderating role of child number between socio-economic status and children’s problem behaviors, which could help parents choose an appropriate parenting style based on their family’s socio-economic status and number of children.

## Data availability statement

The raw data supporting the conclusions of this article will be made available by the authors, without undue reservation.

## Ethics statement

The studies involving human participants were reviewed and approved by the Ethics Committee in the College of Education at Fujian Normal University. Written informed consent to participate in this study was provided by the participants.

## Author contributions

XL contributed to conception and design of the study and organized the database. YL performed the statistical analysis. YZ, YL, and XL wrote the first draft of the manuscript and wrote sections of the manuscript. All authors contributed to the article and approved the submitted version.

## Funding

This project was supported by the National Education Sciences Planning Fund of China (project no. BDA210076).

## Conflict of interest

The authors declare that the research was conducted in the absence of any commercial or financial relationships that could be construed as a potential conflict of interest.

## Publisher’s note

All claims expressed in this article are solely those of the authors and do not necessarily represent those of their affiliated organizations, or those of the publisher, the editors and the reviewers. Any product that may be evaluated in this article, or claim that may be made by its manufacturer, is not guaranteed or endorsed by the publisher.
